# Survey on Parkinson’s Disease Diagnosis Impact: Patients, Caregivers and Health Care Professionals’ Perspectives

**DOI:** 10.3390/jcm13144118

**Published:** 2024-07-14

**Authors:** Stela Dodaj, Margherita Fabbri, Anne Doe de Maindreville, Alexandra Foubert-Samier, Marie-Claire Toussaint, Nicolas Carriere, Jeanne Lopez, Marine Giroud, Virginie Sattler, Angélique Gerdelat, Guillaume Baille, Jean Denis Turc, Christel Barthelemy, Charlotte Scotto d Apollonia, Marie Helene Fabre, Elina Eytier, Claire Thiriez, Frederique Fluchère, Fabienne Ory-Magne

**Affiliations:** 1 Department of Neurology, University Hospital of Toulouse, 31300 Toulouse, France; dodajstela94@gmail.com (S.D.); scottodapollonia.c@chu-toulouse.fr (C.S.d.A.); ory.f@chu-toulouse.fr (F.O.-M.); 2 Department of Clinical Pharmacology and Neurosciences, Clinical Investigation Center CIC1436, Toulouse Parkinson Expert Centre, Toulouse NeuroToul Center of Excellence in Neurodegeneration (COEN), French NS-Park/F-CRIN Network, University of Toulouse 3, CHU of Toulouse, INSERM, 31000 Toulouse, France; 3 Department of Neurology, Hôpital Maison Blanche, 51092 Reims, France; adoedemaindreville@chu-reims.fr (A.D.d.M.); jlopez@chu-reims.fr (J.L.); 4 Institut des Maladies Neurodégénératives, University Bordeaux, CNRS, IMN, UMR5293, 33000 Bordeaux, France; alexandra.foubert@chu-bordeaux.fr; 5 CHU Bordeaux, Service de Neurologie des Maladies Neurodégénératives, IMNc, CRMR AMS, NS-Park/FCRIN Network, 33000 Bordeaux, France; marie-claire.toussaint@chu-bordeaux.fr (M.-C.T.); elina.eytier@chu-bordeaux.fr (E.E.); 6 Bordeaux Population Health Research Center, University Bordeaux, INSERM, BPH, U1219, IPSED, 33000 Bordeaux, France; 7 Department of Neurology, University Hospital of Lille, 59000 Lille, France; 8 Clinique des Minimes, 31200 Toulouse, France; 9 Centre Hospitalier Général, 81000 Albi, France; 10 Clinique des Cèdres, 31700 Cornebarieu, France; 11 Service de neurologie, Hôpital Delafontaine, 93200 St. Denis, France; guillaume.baille@ch-stdenis.fr; 12 Professional Consultant in Neurology, 13500 Martigues, France; jd.turc@wanadoo.fr; 13 Department of Neurology and Parkinson Expert Centre, Caen University-Hospital, 14000 Caen, France; thiriez-c@chu-caen.fr; 14 Service de Neurologie, Neurology Department, CHU Marseille, Hôpital de la Timone, 13000 Marseille, France; frederique.fluchere@ap-hm.fr

**Keywords:** communication, diagnosis, Parkinson’s disease, caregivers

## Abstract

**Background:** The announcement of Parkinson’s disease (PD) diagnosis may provoke negative feelings that impact the ability to cope with the disease and all life changes related to this new condition. There are scarce data on how to improve communication about PD diagnosis and which factors may influence this outcome. **Methods:** We performed **a** national French survey, investigating the diagnosis announcement impact on a large population of people living with PD (PwPD), who recently received the diagnosis (≤1 year since PD diagnosis), and on related caregivers and health care professionals (HCPs), from tertiary and community-based hospitals. **Results:** A total of 397 PwPD (45% female and 82% > 50 years old), 192 caregivers and 120 HCPs (69% neurologists) completed the questionnaire. The diagnosis was not expected by about 60% of PwPD and induced negative feelings in the majority (82%) of them. Negative feelings that PwPD experience in the moment of the diagnosis announcement were related with male gender [OR = 2.034, CI 95% 1.09–3.78; *p* = 0.025] and older age [OR = 1.05, CI 95% 1.01–1.08; *p* = 0.004], while tremor as the first symptom had a threshold significance [OR = 1.78, CI 95% 0.994–3.187; *p* = 0.052]. Half of the PwPD and caregivers considered that they did not receive enough information and one third had a short-term appointment to rediscuss the diagnosis. A total of 82% of PwPD expressed the willingness to have a multidisciplinary follow-up (PD nurse, psychologists). Only 24% of the HCPs had been trained for PD announcement. **Conclusions**: The way a PD diagnosis is delivered represents a pivotal moment in the journey of PwPD and caregivers. This process requires improvement in addressing the gaps expressed by PwPD, caregivers, and HCPs through a participatory approach.

## 1. Introduction

Parkinson’s disease (PD) is the second most common neurodegenerative disorder. Its prevalence is expected to double by 2040 (14 million patients worldwide) [1,2].

Delivering potentially life-changing medical information about a progressive disease such as PD diagnosis is an integrated part of daily work of clinicians and can be difficult for both patients, caregivers and physicians. The announcement of PD diagnosis, which provokes strong negative emotions, may radically change patients’ idea of their future and is a decisive moment, as it can impact the subsequent adaptation of people living with PD (PwPD), their caregiver, and the patient–doctor relationship. The way clinicians deliver the diagnosis can impact patients’ future outcome, such as mental health, treatment adherence, ability to cope with the situation, and decision making [3,4].

A gap has been noticed in the type and amount of information given to patients during the announcement of a chronic neurological condition and that which should be given to properly inform patients [5]. Additionally, the long duration of the prodromal stage, the delay from symptom onset to diagnosis, as well as the diagnostic and prognostic uncertainty, not only affect the quality of life (QoL) and induce stress in patients and their families, but are all also likely to be factors influencing the impact of diagnosis announcement [6,7,8,9].

However, little attention has been paid to how the diagnosis announcement is experienced by PwPD, related caregivers and physicians and what factors influence their experiences. In 2018, a large European survey investigated the impact of PD diagnosis communication on 1775 patients [10] who had received the diagnosis eight years before on approximate average, with symptoms being present from one to more than ten years. A total of 50% reported that the diagnosis was communicated sensitively, and PwPD satisfaction with the diagnostic consultation was positively associated with more sensitive delivery of diagnosis, the usefulness and amount of information provided and time to ask questions. The survey offered relevant data on how to improve PD diagnosis communication but included only the perspective of PwPD and focused on patients who may have received the diagnosis many years before. Of note, the illness of one partner in a couple leads to considerable personal changes and distress for both PwPD and their familial caregivers, causing strain and alterations in their relationship [11]. Couples often feel overwhelmed by the demands of managing the disease and handling daily activities. PD may be related to poor marital adjustment, as couples must navigate shifts in each partner’s role, disruptions in relationship dynamics, and communication challenges [12,13]. Recently, a qualitative study of semi-structured interviews on 15 spousal dyads evaluated the impact of the disease on PwPD and the related familial caregivers’ life and relationship, separately [14]. This study highlighted the difficulties in communication within the couple, the tendency to avoid speaking about the diagnosis and the difficulties experienced to adapt their lives to this new “situation”.

Herein, we report the results of a national survey conducted in France, investigating the impact of diagnosis announcement on a large population of PwPD who recently received the diagnosis, along with caregivers and physicians. Demographic and clinical factors that could have a potential impact on diagnosis announcement were also evaluated as possible suggestions to improve this critical moment.

Of note, the objectives of this study were: (a) analyze current experience of PwPD with the announcement of the disease, (b) highlight the PwPD feelings and identify their needs and desires, (c) identify the feelings and needs of their personal caregivers at the time of the diagnosis, (d) identify the perspectives, obstacles and difficulties faced by professional caregivers, as well as existing needs, and (e) establish an initial amount of data to propose, in the future, possible solutions and ways to make the process of delivering and receiving the PD diagnosis acceptable, comfortable and productive for all parties.

## 2. Methods

### 2.1. Study Design and Participants

This is an observational cross-sectional questionnaire-based study, aiming to investigate the execution and the impact of diagnosis announcement on PwPD, caregivers and health care professionals (HCPs). PwPD who have received the diagnosis within the last year and related caregivers were invited to fill out the online surveys, as well as physicians and HCPs involved in this process, working in both tertiary and community-based hospitals.

### 2.2. Questionnaire Development and Administration

Digital questionnaires were created for a national survey targeting PwPD, related caregivers, physicians (neurologists and non-neurologists) and HCPs. The questionnaires were developed by PD specialized nurses (CB, JL, MCT, MHF) and two psychologists (CS, EE) and neurologists from six PD expert centers (FOM, FF, ADDM, AF, NC, CT) and from general practice (AG, GB, JDT, VS, MG). They were built based on their work experience as well as the needs of patients identified in “*Livre blanc*” of France Parkinson association (https://www.franceparkinson.fr/wp-content/uploads/2023/10/FP_LIVRET_ASSO_COMPLET_250123.pdf, accessed on 1 April 2010). Questions based on the recommendations of the French National Authority for Health (HAS) (Guide for the care pathway—Parkinson’s Disease, February 2012, and its regular updates) have been also integrated. The first version of the questionnaire was elaborated by at least one of each HCP category (PD nurse, psychologist, general and specialized neurologist) who worked together over two meetings. Thereafter, a final meeting with all the HCPs was held to agree on the final questionnaires’ versions that were previously circulated and discussed by email.

The survey link was posted on the France Parkinson Association website and sent to the Association of French-speaking Liberal Neurologists (ANLLF) so that it could be shared to their respective members, clearly specifying the target population and the duration of the survey. Of note, the France Parkinson Association has 10.000 members but it is not possible to know the percentage of PwPD among them; however, it was clearly specified in the questionnaire heading that it should only be filled out by PwPD or a caregiver. The three questionnaires were made digitally available between the 15 February 2021 and the 31 March 2021. In addition, during this period, the neurologists involved in the study also provided it to the recently diagnosed patients and their caregivers, they saw in consultation.

The three questionnaires are structured and self-administered; they include demographic questions, single choice and multiple-choice questions and rating scale questions (Likert scales, numerical scales) (See Supplementary Materials for the complete version of the three questionnaires). Each questionnaire was designed to be completed by participants in approximately 15 min. Participants’ consent was waived as surveys were anonymous and participants gave their approval to fill out the questionnaire online.

## 3. Statistical Analysis

Descriptive statistics were calculated and presented as mean ± standard deviation (SD) or proportions, as appropriate. As our main focus was on the feelings experienced during the PD diagnosis announcement, we elaborated and classified the feelings in two categories: Negative feelings (including the following answers: I was angry; I couldn’t believe; I found it unfair; I was scared; I was scared for my loved ones; I felt there was a misunderstanding; I felt anxious), and Neutral/Positive feelings (I didn’t worry about; I was indifferent; I felt relieved; I was surprised). Univariate logistic regression analysis with the feelings (negative vs. neutral/positive) that patients experienced during the announcement and each following variable as the dependent variable and sex, age at diagnosis (continuous variable), knowing someone suffering from PD (No/Yes), the first motor symptom (tremor/other symptoms), who announced the diagnosis (Neurologist/non-Neurologist), being accompanied during the announcement (yes/no) having received additional information (yes/no) and having a short-term consultation after the announcement (yes/no) as the independent variables were used to calculate unadjusted odds ratio (OR; 95% confidence interval [CI]).

Secondly, all significant factors (*p* ≤ 0.05) were included in a binary logistic regression analysis model to assess the independent association between bad feelings that patients experienced during the PD diagnosis announcement by calculating an adjusted OR (95% CI) for all possible confounding effects. Statistical analysis was conducted using Statistical Package for the Social Sciences (SPSS) 26.0. A *p*-value less than 0.05 was considered statistically significant.

## 4. Results

### 4.1. Patients

Table 1 summarizes demographic features and answers of PwPD. A total of 397 PwPD completed the survey. The average (SD) age was 60.7 (10.0) years, including 55% males. Only 16% of the PwPD were younger than 50 years old at the moment of the diagnosis (23% had less than 55 years and 27% more than 70 years; the others were in between). A total of 39% of participants reported knowing other people suffering from PD the moment of the diagnosis, from which 63% only reported an impact of this fact on the way they see their illness. Tremor was the first symptom presented in 61% of participants, and in 51% of cases the diagnosis was made in less than 12 months from the first motor symptom appearance. In 34% of cases, the diagnosis from the first symptoms was delayed 12 to 24 months, in 7% of cases the delay was 24 to 36 months and in 8% it was more than 36 months. This delay felt long to half of participants (51%). A total of 84% of participants reported that their diagnosis was announced by a neurologist, 9% by their family doctor, 4% by another doctor and the others by another person. Only 40% were expecting the diagnosis of PD.

**Figure 1:**
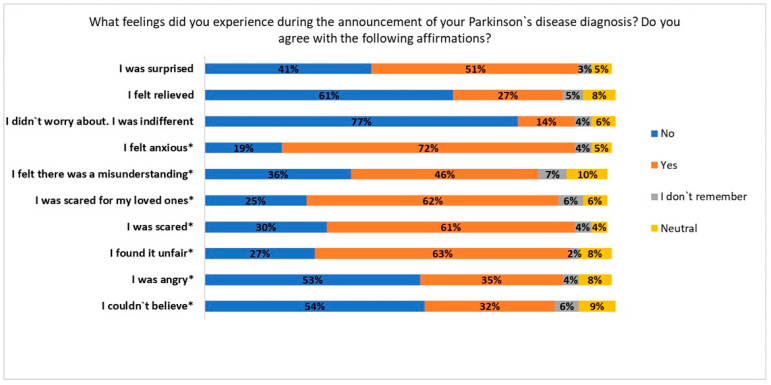
Feelings that patients experienced during the announcement of PD diagnosis (% of respondents). Negative feelings marked with *.

About 82% of patients reported to have experienced negative feelings during the disease announcement (Figure 1). At univariate analysis the following variables resulted to be significant as negative feelings predictors at the moment of the diagnosis announcement: male sex (*p* = 0.015), older age (*p* = 0.003) and tremor as first symptom (*p* = 0.043). When running the logistic regression analysis, the same variables kept significance for negative feelings: male gender; [adjusted OR = 2.034, CI 95% 1.09–3.78; *p* = 0.025], older age [adjusted OR = 1.05, CI 95% 1.01–1.08; *p* = 0.004] while tremor as first symptom had a threshold significance [adjusted OR = 1.78, CI 95% 0.994–3.187; *p* = 0.052] (see Table S1, Supplementary Material). Knowing someone else having PD and being announced by a non-neurologist HCP were not related to bad feelings at diagnosis.

A total of 59% of participants reported that they were alone during the announcement; among the 40% who were accompanied, 92% were with their spouse and the rest by children or other people. When asked if they find the presence of a close relative helpful during the disease announcement, 64% of them answered positively. A total of 51% of participants reported being given enough information during the announcement: they received information about disease and treatment (31%), disease patients’ associations (21%), which health care professionals to contact (29%), care possibilities (15%), while 4% did not remember specifically the information they were provided. Most participants reported that they were not given any leaflet or paper document, were not advised to consult any website about the disease and have not been offered any support from other specialized professionals (nurses, psychologists, etc.), with 80%, 83% and 63% negative answers respectively. When asked if they were invited to participate in an education therapeutical program, 6% of them had no idea what this was, 4% did not remember, 62% reported no as an answer and only 28% reported yes, from which 71% were able to attend. Almost all of those who attended (90%) found it useful or very useful. Only 44% were offered a short-term second consultation at an average delay of 2.9 (1.9) months and most of them (80%) found this waiting time appropriate. A total of 81% of patients found it necessary to have a dedicated PD follow-up consultation with nurses or psychologists, but only 30% reported having benefited from it, finding it little useful in more than half cases (53%).

Finally, when asking PwPD how diagnosis announcement can be improved, having more information about treatment, the disease itself and the progression were the most evoked points (see Figure S1 for detail).

### 4.2. Personal Caregivers

**Figure 2:**
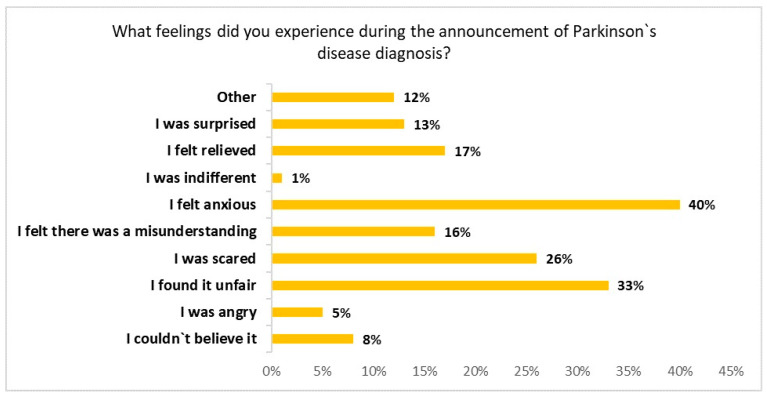
Feelings that caregivers experienced during the announcement of PD diagnosis (% of respondents).

Table 2 summarizes demographic features and answers of caregivers. Figure 2 summarizes the feelings of caregivers at the moment of the diagnosis, highlighting that 40% felt anxiety, about one third found it “unfair” or was scared. A total of 192 personal caregivers = completed the survey, with an average (SD) age of 63.8 (11.1) years and a percentage of 68% females. A total of 72% reported that they knew the disease but 56% of participants reported that they did not know any other PwPD at the moment of the diagnosis. When asked if they were expecting their loved one to be diagnosed with PD, 43% gave a negative answer and 40% gave a positive answer because they themselves had doubted it (40%) or their GP had mentioned it before (14%). More than half reported that they were given enough information, in which 17% found it useful and comprehensive, 20% found it useful but with some missing information, 7% did not understand it, 8% did not find it helpful, 33% reported that they were not given any information but they would have preferred to have, 7% did not need it and 8% did not remember. Most participants reported that they were not advised to consult any website about the disease (84%), join any patients’ association (70%), and have not been invited to participate in an educational therapeutical program (74%) nor any support from other specialized professionals (76%).

When asked about the condition of the announcement, between 66% and 78% of the caregivers found the information clear and understandable, and was given in an appropriate condition and taking enough time to explain (See Figure S2 for details).

### 4.3. Professional Caregivers

Table 3 and Supplementary Figures S3–S8 summarize physicians’ demographic, professional features and answers. A total of 120 physicians completed the survey. The average (SD) age was 44.3 (11.07) years and 65% were females. A total of 69% were neurologists, 21% PD nurses and 7% psychologist/neuropsychologists (3% not specified). About 40% of the physicians see more than 100 PD patients/year and one third has announced this diagnosis to more than 20 patients over the last year. Overall, only 24% were specifically trained on the announcement of PD diagnosis and the possible reactions of patients following the announcement. Even though the majority reported feeling comfortable (66%) and very comfortable (9%) about announcing a chronic disease, when it comes to announcing PD. However, 57% of respondents find it quite difficult and 6% very difficult. Less than half of the physicians and HCPs recommend websites and paper documents (Figure S4) at diagnosis time, but almost all (94%) would consider news ways to announce this diagnosis (Figure S6) and about half of them would consider leaflets, websites (for both PwPD and professionals) and mobile applications, as possibly useful (Figure S7). Among the most important information, professionals indicated non-motor symptoms, motor symptoms, non-pharmacological treatments and care possibilities (Figure S8).

## 5. Discussion

We performed a national survey on a large population of recently diagnosed PwPD, related caregivers and HCPs, to observe PD diagnosis impact throughout these three different perspectives. The diagnosis was not expected by about 60% of PwPD and induced negative feelings in the majority (82%) of them. Male sex and older age were related to worse feelings at diagnosis, while for resting tremor as the first symptom only a trend was noted at regression analysis. Half of the PwPD considered that they did not receive enough information and one third had a short-term appointment to rediscuss diagnosis.

Communication is the key to building a relationship with the patients in all the medical settings with the main goals of creating a good interpersonal relationship, giving the right information, and choosing the proper treatment options. It is a crucial component which values not only the patient’s physical health, but also the patient’s needs, desires, and feelings. In this way, prioritizing their well-being offers them the opportunity to participate in understanding, accepting, and making decisions about their PD diagnosis [4,15].

Interestingly, there are scarce data about the impact of diagnosis announcement among PwPD [10] and even less information on the perspectives of familial caregivers [14] and HCPs regarding this pivotal moment. In 2018, a large survey conducted across 11 European countries reported on PwPD’s perspectives about diagnosis communication. Half of the patients appreciated how the diagnosis was announced, while one-third felt they did not have enough time to ask questions and discuss concerns. When the diagnosis was provided by a specialist, participants reported higher satisfaction with the diagnostic consultation, better sensitivity in communication, more time to ask questions, more useful information, and earlier prescription of medication. Both this previous study and our survey, despite the former being based on different semi-structured interviews, highlight gaps in PD diagnosis announcement and the relationship between communication sensitivity and PwPD’s feelings. Notably, we specifically chose to survey recently diagnosed (<1 year) PwPD to ensure that participants could accurately recall their feelings and thoughts. In contrast, the previous survey, although conducted on a larger population, included PwPD with a much longer delay since diagnosis (a mean of 8 years) and did not incorporate the perspectives of familial and professional caregivers. We believe that our comprehensive approach could better help in understand the critical points of PD diagnosis announcement. Surprisingly, older PwPD seemed to have worse feelings at diagnosis announcement when compared to younger ones, which could suggest that the latter can better cope with diagnosis announcement, even in the case of a neurodegenerative disease. We cannot know if younger PwPD felt less loneliness or had a better social environment, but those factors could be eventually related to less frequency of bad feelings.

When looking at PwPD’s answers, we observed that 60% of them were not expecting to be diagnosed with PD. This finding may explain why the PD diagnosis announcement is often described by patients as a brutal and traumatic event. Furthermore, the uncertainty surrounding a PD diagnosis versus possible differential diagnosis can pose challenges even for physicians to explain the care options and perspectives. In such circumstances, the initial appointment can be unsatisfactory for both parties, which does not bode well for their future relationship. This can result in PwPD distress and dissatisfaction, not only among patients but also among caregivers [10]. These considerations are consistent with the frequency and importance of all the negative feelings observed in our study and with what is classically noted when bad news is announced to any patients [16]. For this reason, honesty and empathy are required, as well as presenting the bad news as a logical sequence of events [3,9,17]. Nowadays, patients and their caregivers seek an honest doctor who demonstrates dedication and empathy, fostering open dialogue by addressing all questions and patient insecurities. This entails clarifying diagnostic information, providing support, and instilling hope for the forthcoming stages of the disease [18].

Almost half of PwPD reported not being given enough information during the announcement of the disease, and this emphasizes the need for improvement in this direction. A large percentage of PwPD had not been provided with any written information in leaflets or paper documents, nor any information about websites about the disease or disease associations. Websites or paper documentation on PD can be complementary and useful tools for diagnosis announcement, especially if this communication is not expected and PwPD and related caregivers have been stunned by it. Those who attended a therapeutic educational program found it useful highlighting the importance of this “support tool” for PD management. Educational programs may have a great impact on social support by providing psychological and emotional care to patients and caregivers but would also relieve the burden of care [19]. Despite its importance, it was only offered to 28% of patients, of which 71% were able to attend. This finding highlights how it can be challenging for PwPD to be included into these programs as they are mostly provided only by expert PD centers, which can be difficult to be reached by PwPD in place of community-based neurologists.

The way of living of PwPD with their diagnosis, at the early stage, seemed to be also influenced by the conditions of the announcement in half cases, as stated by PwPD in the survey. The “condition” of the announcement can imply several aspects, including the empathy of the physicians, the clarity and the amount of the given information and the support offered. This may also suggest that physicians should prioritize providing comprehensive spoken and/or written information to recently diagnosed PD patients. This information may cover the disease itself, its progression, available treatment options, ongoing research, caregiving possibilities, work-related issues, and recommendations for educational programs and support associations.

Despite the challenges physicians face in managing their busy schedules, findings from this survey reveal that PwPD and caregivers express a preference for longer consultations. They value the opportunity to freely ask questions and feel heard during their appointments. Some PwPD even suggest receiving the diagnosis in the presence of a psychologist, letting them meet some other patients and caregivers too, and giving more advice to caregivers on how to follow and deal with a family member suffering from PD. Comprehensive and relevant educational resources can empower PwPD and their caregivers to better understand PD and learn new self-managing strategies as a way to handle living with the disease [9].

Almost half of PwPD and their caregivers in this survey were not offered a second short-term consultation by their physicians. The binary regression analysis did not find the lack of a short-term consultation related to bad feelings at diagnosis announcement, but we cannot exclude that later negative feelings may appear, being related to “loneliness” in the management of a new pathological condition. Planning a second consultation, preferably no longer than 2 months after, is usually recommended, to supplement the information and take care of PwPD’s feelings and uncertainties [8].

Most HCPs find it difficult when it comes to announcing the diagnosis of PD, and this is not related only to lack of experience, but likely to the fact of not receiving any formal training on this. However, we should consider that only 69% of the HCPs were neurologists, and this low proportion could have biased their results. Several strategies for diagnosis communications have been proposed such as didactic approaches, small group discussions, peer role play and standardized patient role play, one to one standardized patient encounters, and they should be implemented since the early medical training [20].

This study, like others in the literature, highlights the importance of a multidisciplinary team in managing PwPD after the diagnosis, as 81% of them expressed the willingness to have a follow-up consultation with specialized health care professionals [21,22]. Indeed, the neurologists and other allied HCPs such as PD specialized nurses, psychologists, neuropsychologists, physiotherapists, etc., should all be considered complementary through the whole new journey that recently diagnosed PwPD are about to start.

Nowadays, studies and guidelines on how to deliver a diagnosis are mainly focused on cancer or fatal illnesses, with very little research on chronic neurological conditions, such as PD. This should encourage us to undertake focus groups and research involving both the giver and the receivers of the PD diagnosis announcement, to foster new strategies and approaches, training programs and recommendations dedicated to PD diagnosis communication. Indeed, the final aim of reaching a better diagnosis communication and care path for the initial disease time, could avoid therapeutic “wandering” and associated impact on QoL and help to establish a personalized care approach.

Our study’s biggest strength is that it is addressed to all three included parties in the diagnosis announcement: PwPD, their personal caregivers and professional caregivers. In addition, it covers a large and considerable sample of recently diagnosed PwPD. In terms of limitations, we have not specifically investigated PwPD clinical or personality traits such as self-compassion abilities or pre-existing anxiety, which have been demonstrated to be related to stigma/psychological stress [23] and resilience factors [24]. Likewise, we had to strike a balance between the length of the questionnaire and the level of detail in the information collected. This includes clinical information about participants’ non-motor symptoms, such as anxiety and depression, which were not collected. However, it should be noted that collecting such non-motor symptoms through an online survey could be challenging, and we preferred to keep the length of the questionnaire to no more than 15 min to increase the likelihood of obtaining a high number of completed surveys. Additionally, the socio-economic characteristics of the participants, which may be confounding factors, were not explored. A final limitation is that no persons with Parkinson’s disease (PwPD) were involved in the questionnaire’s development. This step could be considered for future similar projects.

## 6. Conclusions

The way in which the PD diagnosis is announced is an essential moment in the journey of PwPD and caregivers who are going to co-live with the disease. It has a significant impact on PwPD psychological, emotional and physical well-being. It is also the moment of establishing a relationship with the physician or HCP who made the diagnosis and its announcement, building mutual trust, respect and sensitivity. Our national survey highlights how PD diagnosis announcement can be an unexpected shock for PwPD, with half of them and of caregivers having judged to have not received enough information and most of them expressed the need of a multidisciplinary follow-up. Male, resting tremor at onset and older age are related to negative feelings at diagnosis. A small number of the HCPs received specific training and more than half of them find this announcement quite difficult. We highlight several gaps to improve PD diagnosis announcement that need to be filled, by means of a participatory approach that should include all the actors of this “process”. Our findings could serve as the foundation for a participatory French team to develop tools for announcing a PD diagnosis. These tools could be used by PwPD, caregivers, and HCPs, and might include flyers, scientific and social references/websites, and, last but not least, specialized HCP support specifically offered to recently diagnosed PwPD, over a designated time frame.

## Figures and Tables

**Table 1:** Patients’ clinical features and answers (n = 397).

		Frequency (%)
Gender	Female	45
	Male	55
Age at diagnosis (years)	<50	16
	≥50	84
Did you know other people (family, colleagues) suffering from this disease the moment you were diagnosed?	No	61
	Yes	39
Did this have any impact on the way you see your illness?	No	28
	Yes	63
	I don’t know	9
Which were the first symptoms and problems you had?	Tremor	61
	Other symptoms	39
What was the delay between the first symptoms and the announcement of the disease?	Less than 12 months	51
	12 to 24 months	34
	24 to 36 months	7
	More than 36 months	8
Did this delay feel long?	No	51
	Yes	35
	I don’t remember	14
Who announced the diagnosis of Parkinson’s disease?	Neurologist	84
	Non-Neurologist	16
Were you expecting the diagnosis of Parkinson’s disease?	No	59
	Yes	40
	I don’t remember	1
What feelings did you experience during the announcement of your PD diagnosis?	Negative feelings	82
	Neutral/Positive feelings	18
Were you accompanied during the announcement of the disease?	No	59
	Yes	40
	I don’t remember	1
Who were you accompanied by?	Spouse	92
	Children	4
	Other	4
Were you given enough information during the announcement of the disease?	No	49
	Yes	51
Did they give you any leaflets or paper documents?	No	80
	Yes	16
	I don’t remember	4
Have you been advised to consult any website about the disease?	No	83
	Yes	11
	I don’t remember	6
Have they offered to you any support from other specialized professionals (nurse, psychologist, etc.)?	No	63
	Yes	33
	I don’t remember	4
Have you been invited to participate in a therapeutic educational program?	No	62
	Yes	28
	I don’t know what this is	6
	I don’t remember	4
Were you able to attend?	No	29
	Yes	71
Have you been offered any possibility of involving your caregiver in one of these care options? (support from specialized professionals and/or participation in therapeutic educational program)?	No	55
	Yes	35
	I don’t remember	10
After the announcement of the disease, have you been offered a second consultation to supplement the information?	No	48
	Yes	44
	I don’t remember	8
In your opinion, have the conditions of the disease announcement influenced your way of living with the disease?	No	37
	Yes	50
	I don’t know	13
In your opinion, is it necessary to have a dedicated Parkinson’s disease follow-up consultation with nurses, psychologists, etc.?	No	13
	Yes	81
	I don’t know what this is	6
Do you think that the presence of a close relative (friend, spouse, child, family, etc.) is helpful when the diagnosis is announced?	No	17
	Yes	64
	Neutral	19

**Table 2:** Caregivers’ characteristics and answers (n = 192).

		Frequency (%)
Gender	Female	68
	Male	32
What is your relation with the Parkinson’ disease patient?	Spouse	88
	Children	7
	Friend	1
	Other	4
Are you retired or still working?	Retired	57
	Still working	43
Did you know this disease before?	No	28
	Yes	72
Did you know other people with this disease (family, colleagues, etc.) at the moment of diagnosis?	No	56
	Yes	43
	I don’t know	1
Were you expecting your loved one to be diagnosed with Parkinson’s disease?	No	43
	Yes, I had suspected it	40
	Yes, the doctor had mentioned it	14
	I don’t remember	3
Who announced the diagnosis of Parkinson’s disease?	Neurologist	79
	Non-Neurologist	21
Were you present when the diagnosis was announced?	No	42
	Yes	57
	I don’t remember	1
What feelings did you experience during the announcement of PD diagnosis?	Negative feelings	59
	Neutral/Positive feelings	41
Have you been advised to consult any website about the disease?	No	84
	Yes	9
	I don’t remember	7
Have they advised you to join any patients’ association?	No	70
	Yes	17
	I don’t remember	13
Have you been invited to participate in a therapeutic educational program?	No	74
	Yes	17
	I don’t remember	8
Have they offered to you any support from other specialized professionals (nurse, psychologist, etc.)?	No	76
	Yes	19
	I don’t remember	5
After the announcement of the disease, have you been offered a second consultation to supplement the information?	No	51
	Yes	33
	I don’t remember	16
In your opinion, is it necessary to have a dedicated Parkinson’s disease follow-up consultation with nurses, psychologists, etc.?	No	9
	Yes	84
	I don’t know	7
Do you think that the presence of a close relative (friend, spouse, child, family, etc.) is helpful when the diagnosis is announced?	No	5
	Yes	89
	I don’t know	6

**Table 3:** Professional caregivers’ features (n = 120).

		Frequency (%)
Gender	Female	65
	Male	35
Your profession	Neurologist	69
	PD nurse	21
	Psychologist/Neuropsychologist	7
	Other	3
How many Parkinson’s patients do you see in a year?	Less than 5	2
	5 to 10	3
	11 to 50	24
	51 to 100	30
	More than 100	41
How often do you announce or are involved in the announcement of Parkinson’s disease in one year?	Never	7
	Less than 5 times	12
	5 to 20 times	48
	More than 20 times	33
How long have you been treating patients suffering from Parkinson’s disease?	Less than 5 years	14
	5 to 15 years	45
	More than 15 years	41
Have you received any specific training on the announcement of Parkinson’s disease diagnosis and the possible reactions of patients following the announcement?	No	76
	Yes	24
Overall, how do you feel about the announcing of a chronic disease?	Uncomfortable	3
	Not very comfortable	18
	Comfortable	66
	Very comfortable	9
	Neutral	4
What’s your opinion on announcing the diagnosis of Parkinson’s disease?	No problem at all	1
	Quite easy	27
	Quite difficult	57
	Very difficult	6
	Neutral	9

## Data Availability

Anonymized data of this study will be made available from the corresponding author upon reasonable request.

## Supplementary Materials

The following supporting information can be downloaded at: https://www.mdpi.com/article/10.3390/jcm13144118/s1, Questionnaires, Figures S1: Patients’ suggestions on how could Parkinson’s disease diagnosis announcement be improved, Figure S2: Conditions in which Parkinson’s disease announcement was made. Personal caregivers’ experience, Figure S3: Parkinson’s disease diagnosis announcement. Professional caregivers’ experience, Figure S4: Professional caregivers’ opinions on paper documents and tools used during Parkinson’s disease diagnosis announcement, Figure S5: Work practice experience of professional caregivers in announcing Parkinson’s disease diagnosis, Figure S6: Professional caregivers’ opinions about considering new ways to announce the diagnosis of Parkinson’s disease, Figure S7: Professional caregivers’ opinions on the most useful ways to improve the Parkinson’s disease diagnosis announcement and Figure S8: Professional caregivers’ opinions on improving the information delivered during the Parkinson’s disease diagnosis announcement, Table S1: Factors related to bad feelings at diagnosis announcement. Logistic regression analysis results investigating variables related to bad feeling at diagnosis announcement. Variables used to perform in multiple logistic regression were variables with *p* ≤ 0.05 in univariate logistic regression.
