# Supplementary material for: Survey on Parkinson’s Disease Diagnosis Impact: Patients, Caregivers and Health Care Professionals’ Perspectives
Source: J Clin Med. 2024 Jul 14;13(14):4118. doi: 10.3390/jcm13144118 (PMC11278167; doi:10.3390/jcm13144118)
Supplement: Supplementary file 1 [file jcm-13-04118-s001.zip › Questionnaires, Figures S1, S2, S3, S4, S5, S6, S7 and S8.pdf]

Supplementary Methods

<u>Part I</u>

Questionnaire 1 (Patients)

1. Gender
2. Which is your year of birth?
3. Which year are you diagnosed?
4. Did you know other people (family, colleagues) suffering from this disease the moment you were diagnosed? **No / Yes**

4a. Has this had any impact on the way you see your illness?

- **No**
- **Yes**
- **I don`t know**

5. Which were your first motor symptoms and problems you had?
   - **Tremor**
   - **Other symptoms (Stiffness, slowness, walking difficulties, other)**

6. Which was the delay between the first symptoms and the announcement of the disease?
   - **Less than 12 months**
   - **12 to 24 months**
   - **24 to 36 months**
   - **more than 36 months**

6a. Did this delay feel long?

- **No**
- **Yes**
- **I don`t remember**

7. What have you felt during this waiting time? (more than one answer possible)
   - **Anxiety/Worry/Stress**
   - **Impatience**
   - **Indifference**
   - **Anger**
   - **Despair**
   - **I don`t remember**
   - **Other**
8. Who has announced the diagnosis of Parkinson Disease?
   - **Neurologist**
   - **Non-Neurologist**
9. Were you expecting the diagnosis of Parkinson`s disease?
   - **No**
   - **Yes**
   - **I don`t remember**

9a. If `Yes`, please specify:

- **I had doubted it**
- **My doctor had already discussed it with me**
- **I had talked about it with my family and friends**

10. What feelings did you experience during the announcement of your Parkinson`s disease diagnosis? Do you agree with the following affirmations?

- I couldn`t believe **No/ Yes/ I don`t remember/ Neutral**
- I was angry **No/ Yes/ I don`t remember/ Neutral**
- I found it unfair **No/ Yes/ I don`t remember/ Neutral**
- I was scared **No/ Yes/ I don`t remember/ Neutral**
- I was scared for my loved ones **No/ Yes/ I don`t remember/ Neutral**
- I felt there was a misunderstanding **No/ Yes/ I don`t remember/ Neutral**
- I felt anxious **No/ Yes/ I don`t remember/ Neutral**
- I didn`t worry about. I was indifferent **No/ Yes/ I don`t remember/ Neutral**
- I felt relieved **No/ Yes/ I don`t remember/ Neutral**
- I was surprised **No/ Yes/ I don`t remember/ Neutral**

11. Were you accompanied during the announcement of the disease?

- **No**
- **Yes**
- **I don`t remember**

11a. Who you were accompanied with?

- **Spouse**
- **Children**
- **Other**

12. Were you given enough information during the announcement of the disease? **No/ Yes**

12a. What topics were you informed about? (more than one answer possible)

- **Disease and treatment**
- **Disease associations**
- **Which professionals to contact**
- **The care possibilities**
- **I don`t remember**

13. Did they give you any leaflets or paper documents?

- **No**
- **Yes**
- **I don`t remember**

14. Have you been advised to consult any website about the disease?

- **No**
- **Yes**
- **I don`t remember**

15. Have they proposed to you any support from other specialized professionals (nurse, psychologist, etc.)?

- **No**
- **Yes**
- **I don`t remember**

15a. Have you done that? **No/ Yes**

16. Have you been proposed to participate in a therapeutic educational program?
- **No**
- **Yes**
- **I don`t know what this is**
- **I don`t remember**

16a. Were you able to attend? **No/ Yes**

16b. How did you find the therapeutic educational program?

- **Little useful**
- **Useful**
- **Very useful**

17. After the announcement of the disease, have you been proposed a second consultation to complete the information?
- **No**
- **Yes**
- **I don`t remember**

17a. How long after? ______(months)

17b. Was this waiting time appropriate?
- **No**
- **Yes**
- **I don`t remember**

18. In your opinion, have the conditions of disease announcement influenced your way of living with the disease??
- **No**
- **Yes**
- **I don`t know**

19. In your opinion, is it necessary to have a dedicated Parkinson`s disease follow-up consultation with nurses, psychologists, etc.?
- **No**
- **Yes**
- **I don`t know what this is**

19a. Have you benefited from it? **No/ Yes**

20. Do you think that the presence of a close relative (friend, spouse, child, family, etc.) is helpful when the diagnosis is announced?
- **No**
- **Yes**
- **Neutral**

21. In your opinion, how could Parkinson`s disease diagnosis announcement be improved? (more than one answer possible):

- Giving more information about the disease
- Giving more information about the disease progression
- Giving more information about the treatment
- Giving more information about the research on the disease
- Giving more information about care possibilities and process
- Giving more information about work-related issues
- Extending the consultation time of the disease announcement
- More availability to answer questions
- Redoing the announcement with the caregiver if he/she hasn`t been present
- Announcing the diagnosis to the patient/caregiver in the presence of a psychologist
- Advising caregivers on how to support the patient who has just been diagnosed
- Allowing to meet other patients/caregivers
- Other

Questionnaire 2 (Personal caregivers)

1. Gender
2. Which is your relation with the Parkinson`s disease patient?
- **Spouse**
- **Child**
- **Friend**
- **Other**

7. Are you retired or still working?
- **Retired**
- **Still working**

8. Did you know this disease before?
- **No**
- **Yes**

8a. How have you known it? (more than one answer possible)

- **You had read or seen some reports about it**
- **Someone else you know had this disease**
- **Someone has talked to you about it**
- Other

9. Did you know other people with this disease (family, colleagues, others…) at the moment of diagnosis?
- **No**
- **Yes**
- **I don`t know**

10. Were you expecting your loved one to be diagnosed with Parkinson's disease?
- **No**
- **I had doubted it**
- **The doctor had mentioned it**
- **I don`t remember**

11. Who has announced the diagnosis of Parkinson`s disease?

- Neurologist
- Non-Neurologist

12. Were you present when the diagnosis was announced?
- **No**
- **Yes**
- **I don`t remember**

13. What feelings did you experience during the announcement of PD diagnosis? (more than one answer possible)
- **I couldn`t believe it**
- **I was angry**
- **I found it unfair**
- **I was scared**
- **I found there was a misunderstanding**
- **I felt anxious**
- **I was indifferent**
- **I felt relieved**
- **I was surprised**
- **Other**

14. If there was a doctor who made the diagnosis, has he given enough information about the disease?
- **Yes, I found it useful and comprehensive**
- **I found it useful but some information was missing**
- **Yes, but i haven`t understood it all**
- **Yes, but it didn`t help/ No, but i didn`t need**
- **No, but i would have preferred to have**
- **I don`t remember**

15. Have you been advised to consult any website about the disease?
- **No**
- **Yes**
- **I don`t remember**

16. Have they advised you to join any patients` association?
- **No**
- **Yes**
- **I don`t remember**

17. Have you been proposed to participate in a therapeutic educational program?
- **No**
- **Yes**
- **I don`t remember**

18. Have they proposed to you any support from other specialized professionals (nurse, psychologist, etc.)?
- **No**
- **Yes**
- **I don`t remember**

19. After the announcement of the disease, have you been proposed a second consultation to complete the information?

- **No**
- **Yes**
- **I don`t remember**

20. What is your opinion on the conditions of the disease announcement?

20a. The announcement was made in a proper place and a calm environment **No/ Yes/ I don`t remember/ Neutral**

20b.The information was clear and understandable environment **No/ Yes/ I don`t remember/ Neutral**

20c.The doctor took all the time needed environment **No/ Yes/ I don`t remember/ Neutral**

20d. I was listened by my doctor environment **No/ Yes/ I don`t remember/ Neutral**

20e. I could ask questions environment **No/ Yes/ I don`t remember/ Neutral**

20f. The doctor made sure I had understood the information he had just given environment **No/ Yes/ I don`t remember/ Neutral**

20g. The doctor gave us a lot of information environment **No/ Yes/ I don`t remember/ Neutral**

21. In your opinion, is it necessary to have a dedicated Parkinson`s disease follow-up consultation with nurses, psychologists, etc.?

- **No**
- **Yes**
- **I don`t know**

22. Do you think that the presence of a close relative (friend, spouse, child, family, etc.) is helpful when the diagnosis is announced??

- **No**
- **Yes**
- **I don`t know**

Questionnaire 3 (Professional caregivers)

1. Gender
2. How old are you?
3. Your profession?

- **Neurologist**
- **PD nurse**
- **Psychologist/Neuropsychologist**
- **Other**

4. How many Parkinson`s patients do you see in a year?

- **Less than 5**
- **5 to 10**
- **11 to 50**
- **51 to 100**
- **More than 100**

5. How often do you announce or are involved in the announcement of Parkinson`s disease in one year?

- **Never**
- **Less than 5 times**
- **5 to 20 times**
- **More than 20 times**

6. How long have you been treating patients suffering from Parkinson's disease?

- **Less than 5 years**
- **5 to 15 years**
- **More than 15 years**

7. Have you received any specific training on the announcement of Parkinson`s disease diagnosis and the possible reactions of patients following the announcement?

- **No**
- **Yes**

8. Overall, how do you feel about announcing a chronic disease?

- **Uncomfortable**
- **Not very comfortable**
- **Comfortable**
- **Very comfortable**
- **Neutral**

9. What` s your opinion on announcing the diagnosis of Parkinson`s disease?

- **No problem at all**
- **Quite easy**
- **Quite difficult**
- **Very difficult**
- **Neutral**

10. Regarding your experience in announcing the diagnosis of Parkinson`s disease, what`s your opinion about the following statements:

- In most cases, the announcement is well received by patients **Strongly disagree/ Disagree/ Agree/ Strongly agree/ Neutral**
- In most cases, the announcement is badly received by patients **Strongly disagree/ Disagree/ Agree/ Strongly agree/ Neutral**
- The announcement is mostly a shock for patients, but they adapt over time **Strongly disagree/ Disagree/ Agree/ Strongly agree/ Neutral**
- In general, healthcare professionals receive little training in the diagnosis announcement of chronic diseases **Strongly disagree/ Disagree/ Agree/ Strongly agree/ Neutral**

11. Regarding your experience in announcing the diagnosis of Parkinson`s disease, what`s your opinion in the following statements about paper documents and tools used:

- Paper documents explaining the disease to patients are easily available **Strongly disagree/ Disagree/ Agree/ Strongly agree/ Neutral**
- There are several websites I recommend to patients in order to get informed **Strongly disagree/ Disagree/ Agree/ Strongly agree/ Neutral**
- I formally advise patients against seeking information on the Internet **Strongly disagree/ Disagree/ Agree/ Strongly agree/ Neutral**

12. Regarding your experience in announcing the diagnosis of PD, what`s your opinion about the following statements about your work practice:

- I don't have as much time as I'd like to have when I'm announcing a Parkinson's disease **Strongly disagree/ Disagree/ Agree/ Strongly agree/ Neutral**
- I don't have enough time to explain the disease, but I refer patients to other resources that can answer their questions **Strongly disagree/ Disagree/ Agree/ Strongly agree/ Neutral**
- I always suggest a consultation with a psychologist following the announcement **Strongly disagree/ Disagree/ Agree/ Strongly agree/ Neutral**
- I systematically propose a consultation with a PD specialized nurse after the announcement consultation **Strongly disagree/ Disagree/ Agree/ Strongly agree/ Neutral**
- I systematically propose a consultation with another healthcare professional following the announcement consultation **Strongly disagree/ Disagree/ Agree/ Strongly agree/ Neutral**
- I always give the contact details of France Parkinson association (or another association) **Strongly disagree/ Disagree/ Agree/ Strongly agree/ Neutral**
- I systematically suggest seeing patients again within a short timeframe (<1 month) to rediscuss the announcement **Strongly disagree/ Disagree/ Agree/ Strongly agree/ Neutral**
- I systematically suggest seeing patients again within a short timeframe (<3 month) to rediscuss the announcement **Strongly disagree/ Disagree/ Agree/ Strongly agree/ Neutral**
- I systematically suggest a consultation with the nearest Parkinson educational therapeutic program team to join the program **Strongly disagree/ Disagree/ Agree/ Strongly agree/ Neutral**

13. How would you consider some new ways to announce the diagnosis of Parkinson`s disease?

- **Useless**

- **Little useful**

- **Useful**

- **Very useful**

- **Neutral**

14. In your opinion, which would be the most useful way to improve the announcement of PD diagnosis?

- Leaflets to be given to the patients **Completely useless/ Useless/ Little useful/ Useful/ Very useful/ Neutral**

- Paper documents intended to be presented to the patient by the professional **Completely useless/ Useless/ Little useful/ Useful/ Very useful/ Neutral**

- Websites that can be directly used by the patients **Completely useless/ Useless/ Little useful/ Useful/ Very useful/ Neutral**

- Website for use by the professionals with the patient **Completely useless/ Useless/ Little useful/ Useful/ Very useful/ Neutral**

- Mobile and/or tablet application **Completely useless/ Useless/ Little useful/ Useful/ Very useful/ Neutral**

- Reference website on PD advertising for professionals only **Completely useless/ Useless/ Little useful/ Useful/ Very useful/ Neutral**

15. In your opinion, which is the most important information that needs to get improved: **Completely useless/ Useless/ Little useful/ Useful/ Very useful/ Neutral**

- Information about the epidemiology of the disease

- Information about the pathophysiology of the disease

-Information about motor symptoms

- Information about non motor symptoms

- Information about pharmacological treatments for motor symptoms

- Information about pharmacological treatments for non-motor symptoms

- Information about nonpharmacological treatments

- Information about disease progression

- Information about research on this disease

- Information about care options (psychological, social etc.)

- Information about work related issues

- Information on possible reactions following the announcement of a chronic illness

Supplementary Graphs

A) Patients

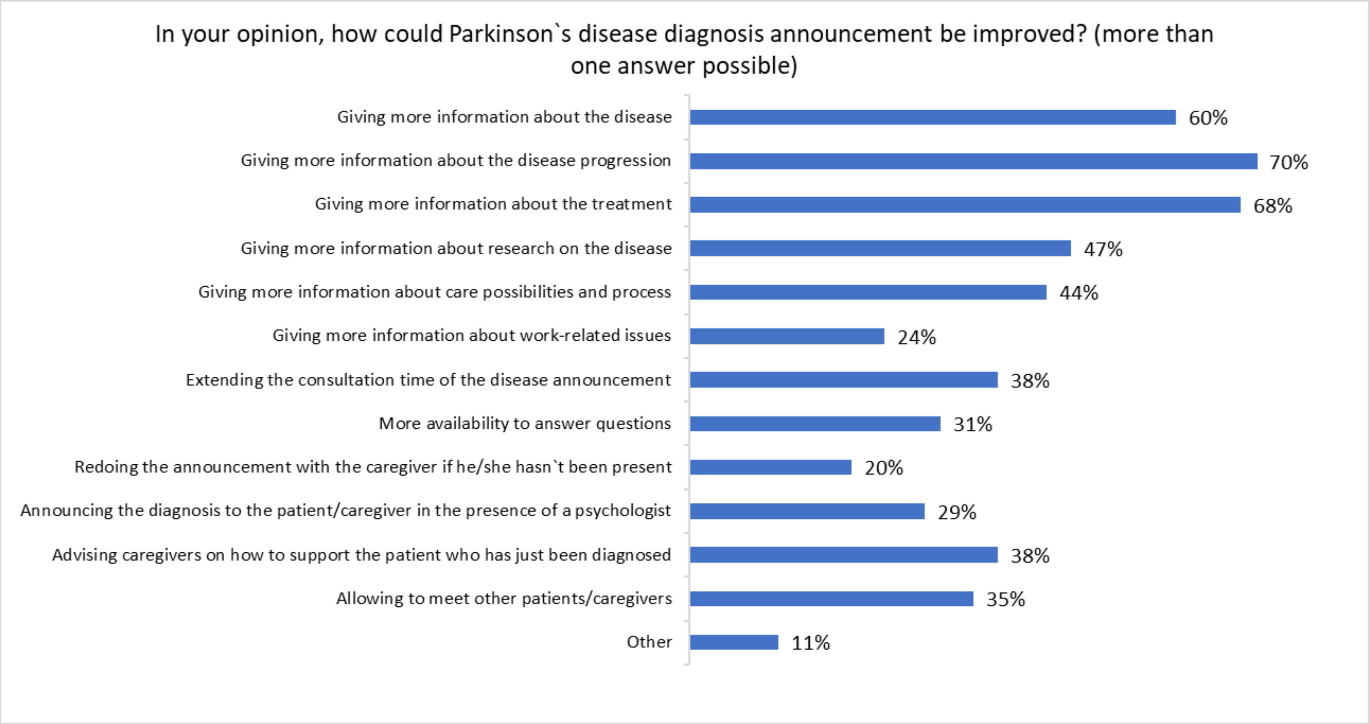

Figure S1: Patients` suggestions on how could Parkinson`s disease diagnosis announcement be improved

B) Personal caregivers

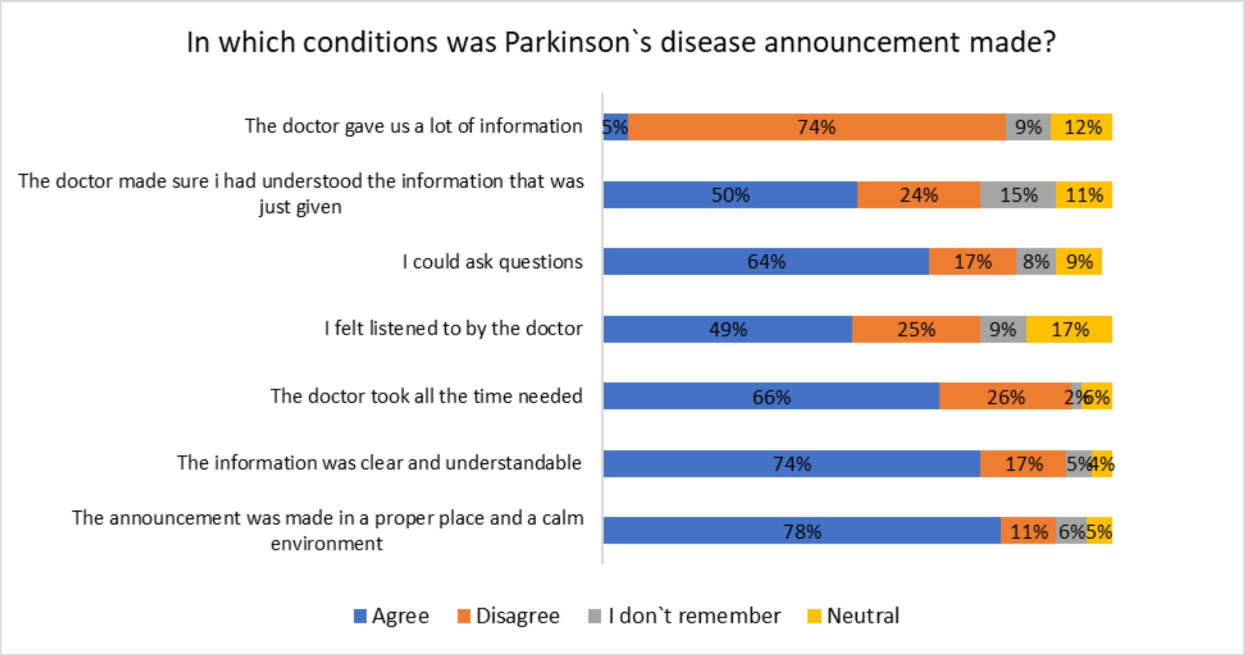

Figure S2: Conditions in which Parkinson`s disease announcement was made. Personal caregivers` experience.

C) Professional caregivers

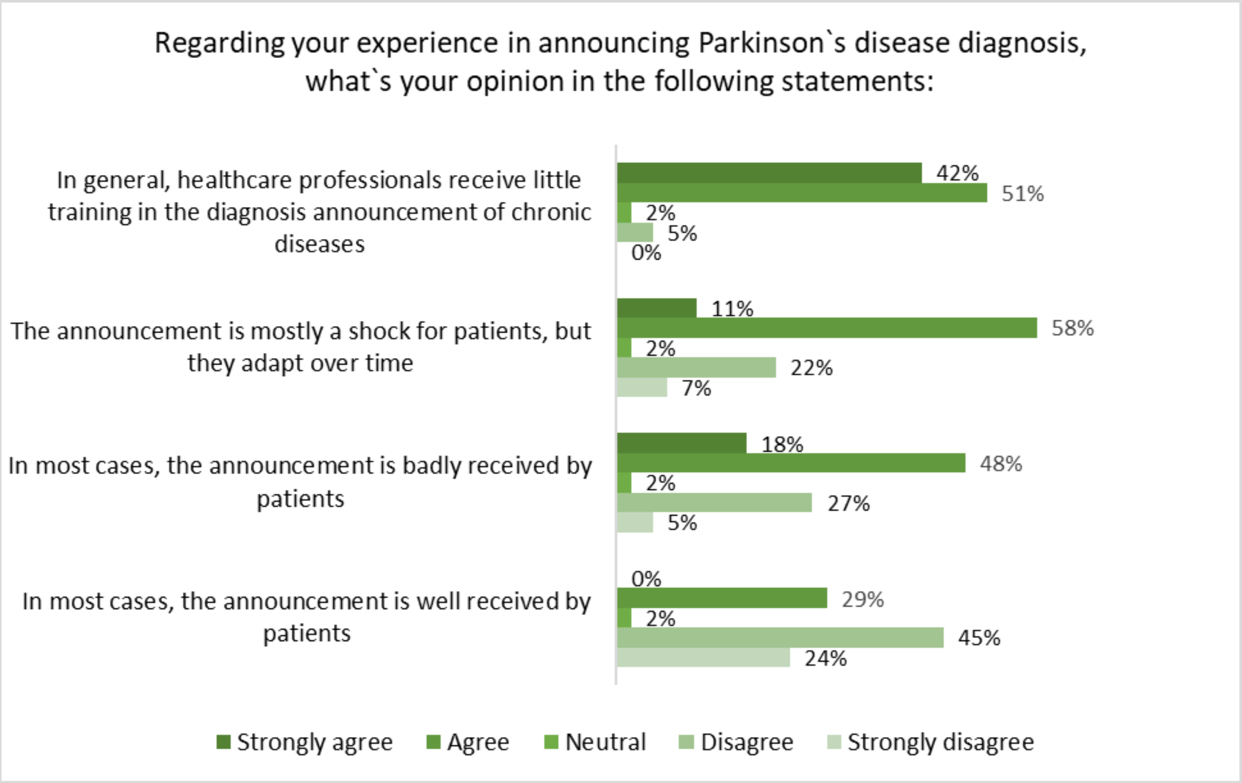

Figure S3: Parkinson`s disease diagnosis announcement. Professional caregivers` experience.

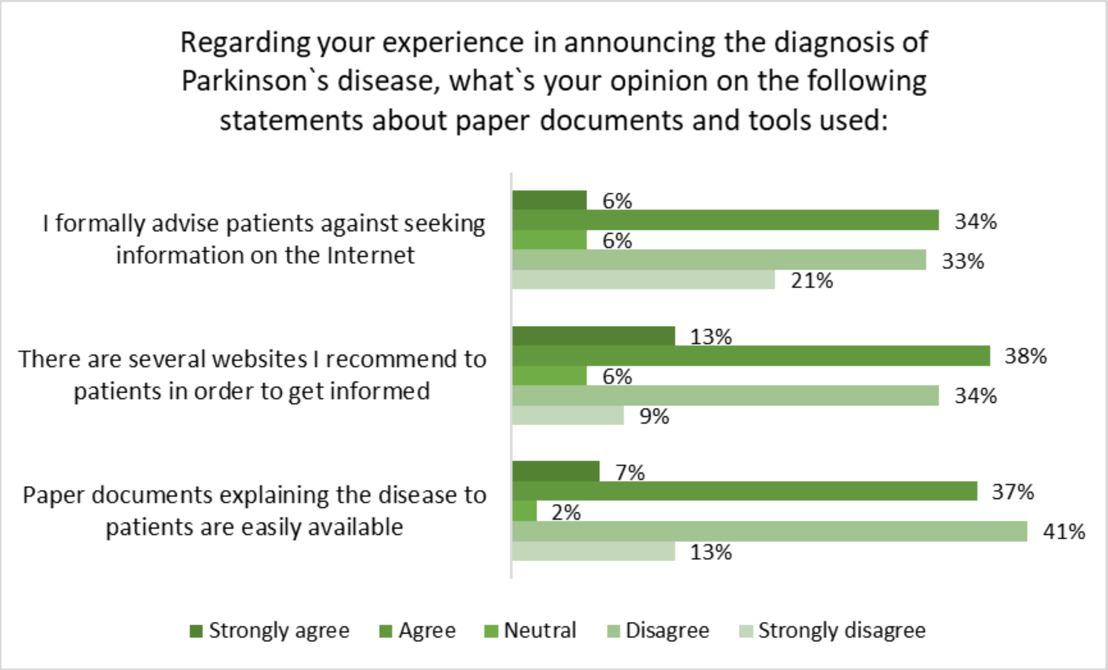

Figure S4: Professional caregivers` opinions on paper documents and tools used during Parkinson`s disease diagnosis announcement.

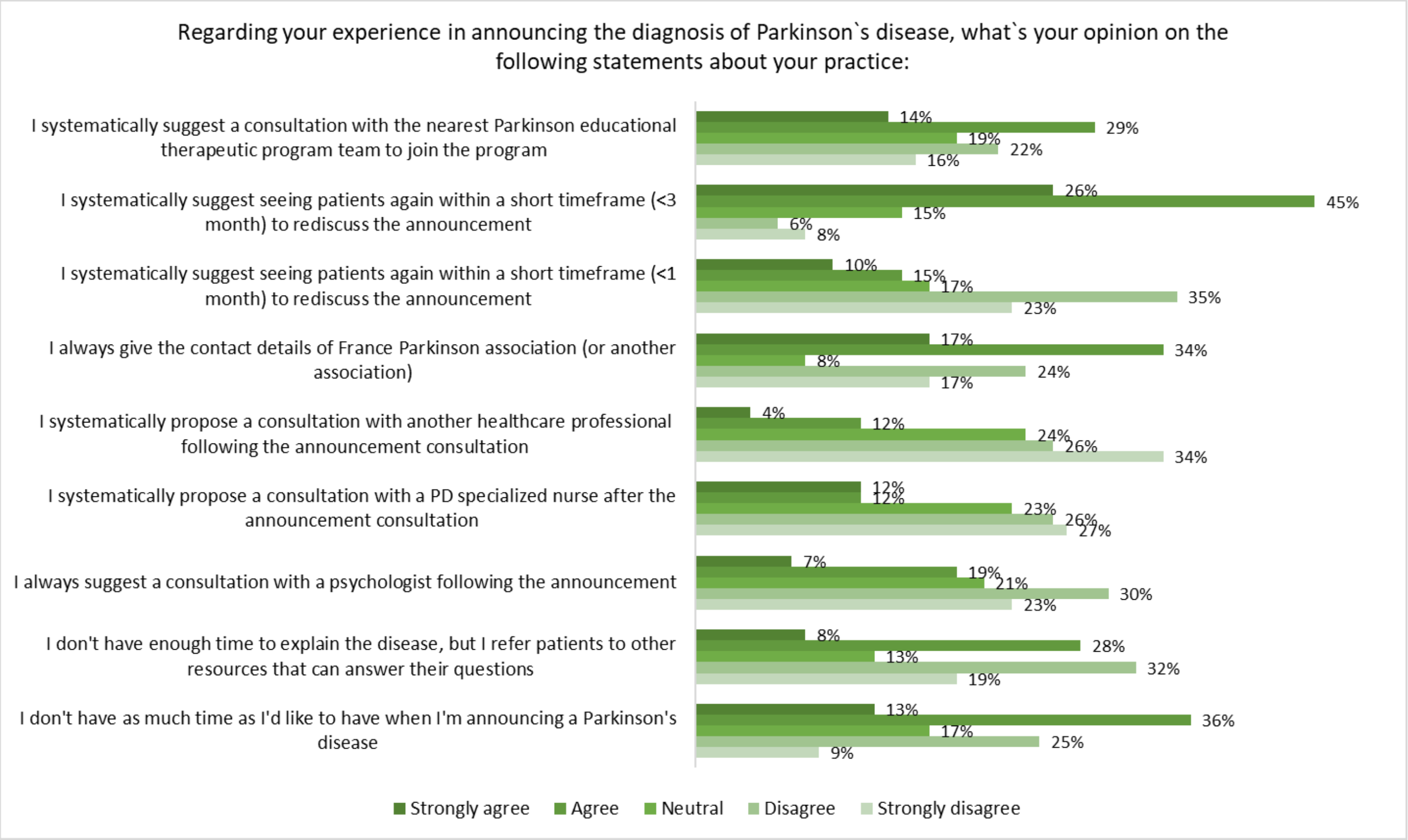

Figure S5: Work practice experience of professional caregivers in announcing Parkinson`s disease diagnosis

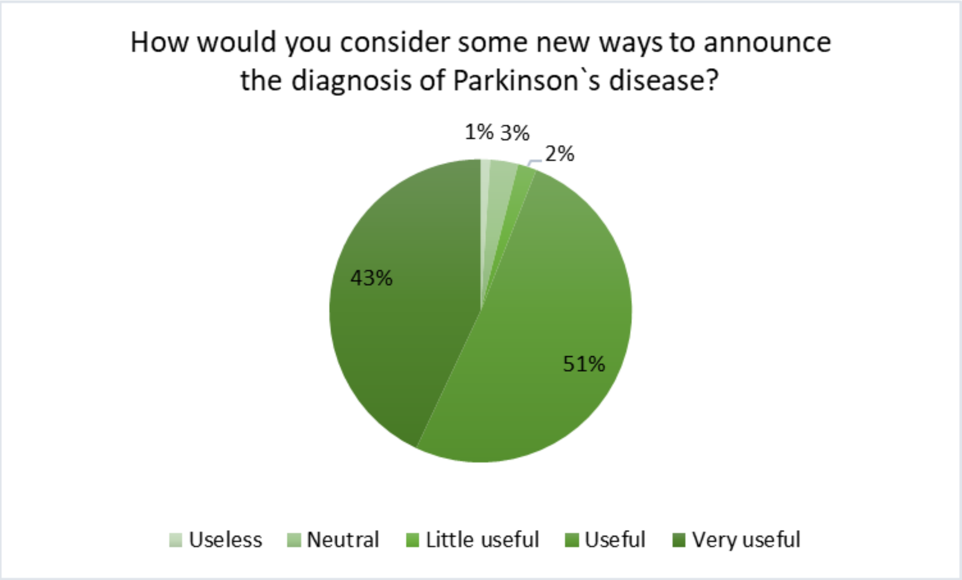

Figure S6: Professional caregivers` opinions about considering new ways to announce the diagnosis of Parkinson`s disease

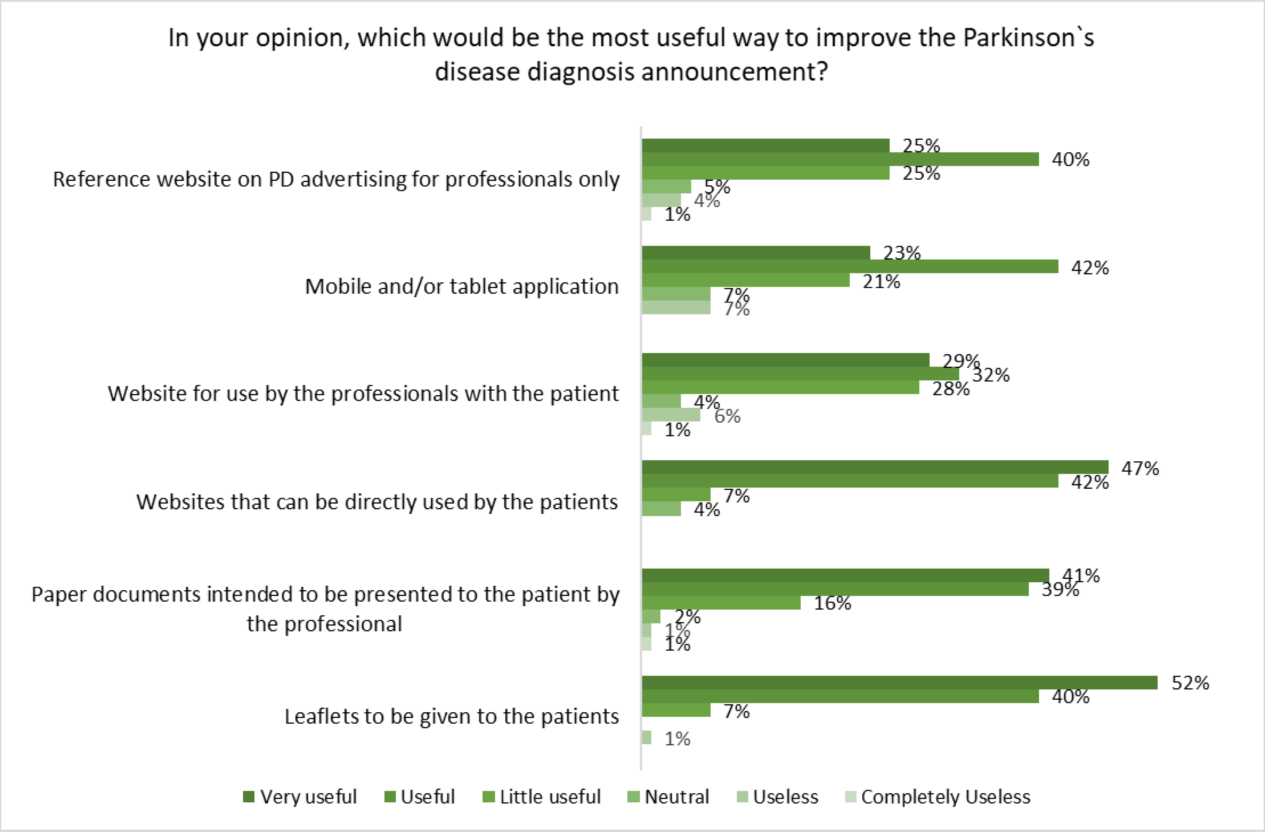

Figure S7: Professional caregivers` opinions on the most useful ways to improve the Parkinson`s disease diagnosis announcement

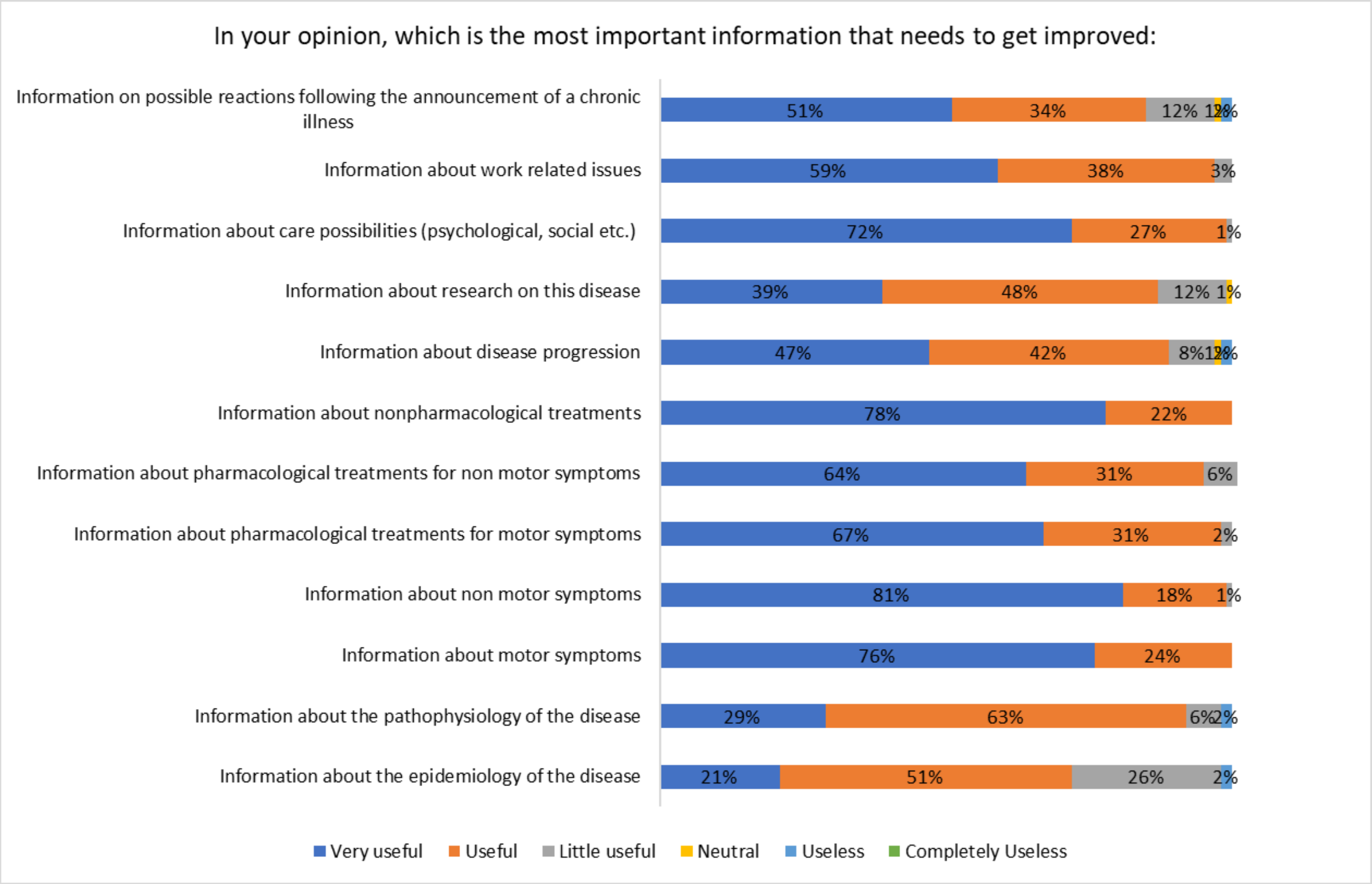

Figure S8: Professional caregivers` opinions on improving the information delivered during the Parkinson`s disease diagnosis announcement
