# Supplementary material for: Survey on Parkinson’s Disease Diagnosis Impact: Patients, Caregivers and Health Care Professionals’ Perspectives
Source: J Clin Med. 2024 Jul 14;13(14):4118. doi: 10.3390/jcm13144118 (PMC11278167; doi:10.3390/jcm13144118)
Supplement: Supplementary file 1 [file jcm-13-04118-s001.zip › Supplementary Table S1-resubmitted.pdf]

	Unadjusted			Adjusted		
	OR	CI95%	P value	OR	CI95%	P value
Gender	2.008	1.142-3.530	0.015	2.034	1.09-3.78	0.025
Age	1.046	1.015-1.077	0.003	1.05	1.01-1.08	0.004
Did you know other people before	0.777	0.448-1.348	0.369	/	/	/
Tremor as first symptoms	1.721	1.018-2.909	0.043	1.780	0.994-3.187	0.052
Diagnosis announced by a neurologist (vs. non-neurologist)	0.659	0.298-1.459	0.304	/	/	/

Table S1. **Factors related to bad feelings at diagnosis announcement**. Logistic regression analysis results investigating variables related to bad feeling at diagnosis announcement. Variables used to perform in multiple logistic regression were variables with $p \leq 0.05$ in univariate logistic regression.
